# Microbial Spectra and Clinical Outcomes from Endoscopically Drained Pancreatic Fluid Collections: A Descriptive Cohort Study

**DOI:** 10.3390/antibiotics11030420

**Published:** 2022-03-21

**Authors:** Viktoria Hentschel, Benjamin Walter, Noemi Harder, Frank Arnold, Thomas Seufferlein, Martin Wagner, Martin Müller, Alexander Kleger

**Affiliations:** 1 Department of Gastroenterology, Clinic of Internal Medicine 1, University Hospital of Ulm, 89081 Ulm, Germany; viktoria.hentschel@uniklinik-ulm.de (V.H.); benjamin.walter@uniklinik-ulm.de (B.W.); noemi.harder@uni-ulm.de (N.H.); frank.arnold@uni-ulm.de (F.A.); thomas.seufferlein@uniklinik-ulm.de (T.S.); martin.wagner@uniklinik-ulm.de (M.W.); martin.mueller@uniklinik-ulm.de (M.M.); 2 Interdisciplinary Endoscopy Unit, University Hospital of Ulm, 89081 Ulm, Germany

**Keywords:** pancreatitis, acute necrotizing, pancreatic pseudocyst, bacterial infections, mycoses, anti-infective agents

## Abstract

Pancreatic pseudocyst (PC) and walled-off necrosis (WON) are dreaded complications of acute pancreatitis. Standard therapy consists of endoscopic ultrasound-guided transmural placement of stents to expedite resolution through internal drainage of fluids or necrotic material. Either double pigtail plastic stents (DPPS) or lumen-apposing metal stents (LAMS), or a combination of both, are available for this purpose. The objective of this study was to examine the impact of different stent types on infection rates in addition to clinical outcome measures such as periprocedural adverse events. We conducted a retrospective study comprising 77 patients who had undergone endoscopic drainage for PC or WON in a pancreatitis tertiary referral center. Analysis revealed that both bacterial and fungal infections occurred more frequently in patients treated with LAMS with or without DPPS compared to DPPS only. The use of antibiotics and antimycotics followed the same pattern. Furthermore, a prolonged length of hospital stay and a higher likelihood of transfer to an intermediate care unit were observed in patients with LAMS with or without DPPS. These differences were eliminated if only WON patients were analyzed. Our data imply that the clinical course is primarily influenced by the complexity of the pancreatic fluid collection (PFC) itself rather than the stent type. Prospective large-scale cohort studies are mandatory to underpin these findings.

## 1. Introduction

Worldwide, acute pancreatitis is recognized as a serious condition, calling for immediate medical attention due to its potential to devolve into a critical and, at times fatal, disease course [1,2]. The global age-standardized incidence is currently estimated to be 20.6 per 100,000 of the general population [3]. Inflammatory destruction of the pancreatic parenchyma is followed by a massive release of proteolytic and lipolytic enzyme precursors as well as proinflammatory cytokines and vasoactive peptides, accounting for uncontrolled tissue degradation, systemic inflammation and generalized extravasation of body fluids due to endothelial leakage [4,5]. In addition to defining clinical criteria obligatory for diagnosing and grading disease severity, the 2012 revised Atlanta classification system specifies local complications and pancreatic fluid collections (PFC) occurring during acute pancreatitis. Based on the emergence of necrotic collections, the algorithm discriminates acute interstitial and necrotizing pancreatitis. Demarcated collections are termed either acute peripancreatic fluid collection or acute necrotizing collection if they form within 4 weeks from disease onset. In the later time course, encapsulation may cause permanent retention of collections, either termed pancreatic pseudocyst (PC) in the case of pancreatic fluid retention or walled-off necrosis (WON) in the case of encapsulated necrotic tissue, both being potentially amenable to endoscopic drainage [6].

Over the past years, endoscopic ultrasound-guided stent-assisted transmural drainage (EUS-TD) has become the first-line therapeutic approach to evacuate persistent symptomatic sterile or infected collections, alleviate the accumulation of digestive secretions from duct disruption and thus prevent progressive enzymatic liquefaction of pancreatic and neighboring tissue [7,8]. In comparison to percutaneous or surgical drainage, EUS-TD attained similar technical success rates while at the same time being associated with decreased invasiveness, lower reintervention and morbidity rates and shorter in-patient stays [9]. A fundamental condition for safely and effectively performing EUS-TD with any stent type is the presence of a wall solid enough to support stable stent positioning. Either double pigtail plastic stents (DPPS) or lumen-apposing metal stents (LAMS) can be used to promote internal drainage of PFC. DPPS are advantageous in that they are relatively cost-effective and easy both to place and retrieve but, owing to their small caliber, are predisposed to preterm obturation and migration. By contrast, LAMS are characterized by large-bore caliber sizes with extended patency and offer unimpeded access to the cavity for direct necrosectomies. However, stent migration and bleeding have been described as relevant adverse events [10,11].

The impact of stent type selection on basic clinical trial endpoints as well as directly procedure-related short- and long-term complications has inspired many observational studies and systematic reviews. However, the overall prognosis of patients may be substantially impaired by systemic bacterial and fungal infection. The natural course of acute severe pancreatitis is characterized by a linear relationship between duration and bacterial infection rates, rising up to 39–60% [12,13]. Literature suggests that extensive necrosis, affecting > 30% of pancreatic tissue, potentiates the risk of bacterial infection, which again is associated with increased morbidity and mortality [12,14]. However, the role of antibiotic prophylaxis remains controversial, as several studies investigating its impact on mortality have failed to demonstrate a definitive survival benefit [15,16,17,18,19]. Thus, current guidelines generally do not recommend prophylactic administration of antibiotics [20,21].

Similarly, fungal infections demand close attention and prompt initiation of concomitant antifungal therapy in case of positive culture results or septic deterioration not responding to antibiotics [22]. Exposure to broad-spectrum antibiotics, indwelling synthetic devices and repeated invasive interventions of the gastrointestinal tract have each been identified as high-risk settings, which is why the administration of antifungal prophylaxis was proposed by several authors [23].

This retrospective cohort study aims at comparing DPPS and LAMS, used either separately or in combination, for endoscopic drainage of post-pancreatitis PC or WON, respectively. Besides general clinical endpoints as well as procedure- and disease-specific complications, we were interested in exploring whether stent material properties may affect the risk for bacterial and fungal infections of the PFC. In addition, we reviewed the frequency of antibiotic and antimycotic use, separated by stent type, and provided an illustration of the microbial spectrum of distinct PFC types together with antimicrobial susceptibility testing of the most relevant pathobionts.

## 2. Results

### 2.1. Epidemiological and Clinical Characteristics of the Study Population

**Figure 1:**
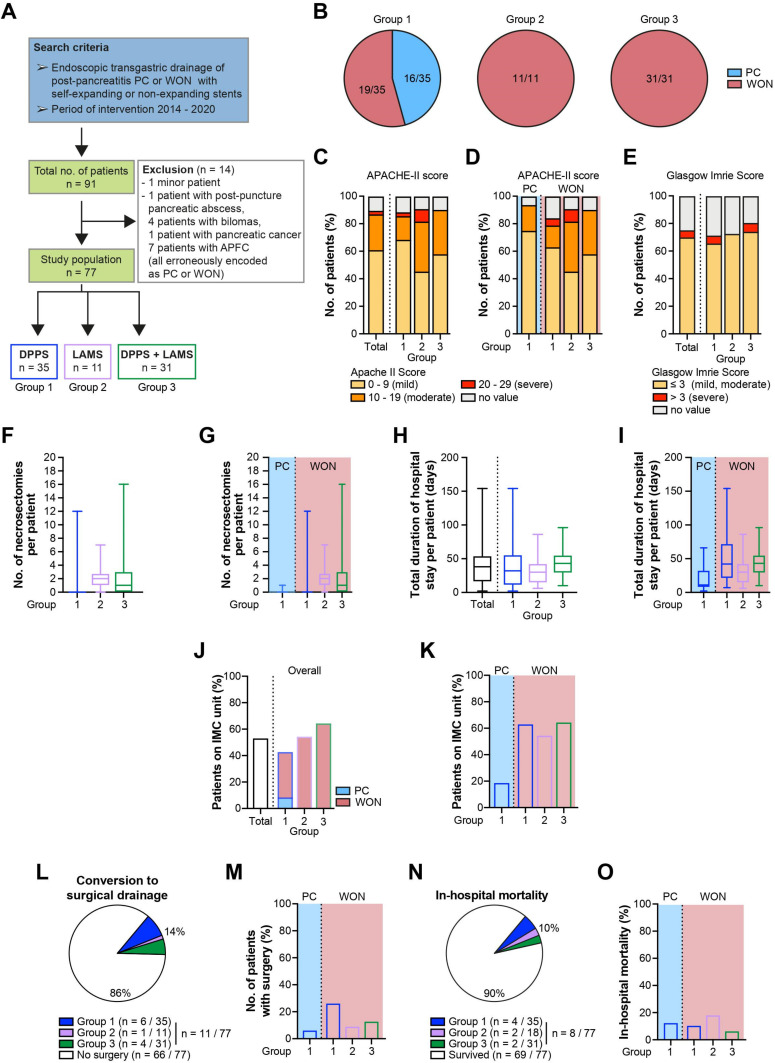
(**A**) Flowchart of study population. (**B**) Composition of stent groups according to PFC type. Grading of disease severity according to APACHE-II score related to overall study population and stent group (**C**) and PFC type (**D**). (**E**) Grading of disease severity according to Glasgow Imrie score related to overall study population and stent group. (**F**) Number of necrosectomies performed per stent group. (**G**) Number of endoscopic necrosectomy sessions separated by PFC type. Total duration of in-hospital stay per patient related to overall study population and stent group (**H**) and PFC type (**I**). Proportion of patients requiring medical support at IMC level related to overall study population and stent group (**J**) and PFC type (**K**). Proportion of patients undergoing surgical PFC drainage related to overall study population and stent group (**L**) and PFC type (**M**). Hospital mortality rates related to overall study population and stent group (**N**) and PFC type (**O**).

A total of 77 patients (54 male, 23 female) undergoing treatment were analyzed, including 35 with DPPS only (group 1), 11 with LAMS only (group 2) and 31 with a combination of both stent types (group 3) (Figure 1A). While group 1 was mixed, including both patients with PC and WON (16/35, 46% and 19/35, 54%, respectively), group 2 and group 3 were composed of patients with WON only (Figure 1B). A comprehensive overview of all clinical outcome parameters is displayed in Table 1.

The mean age (±standard deviation) of patients was 55 ± 17 years with an equal distribution among all groups (group 1: 55 ± 14, group 2: 56 ± 17, group 3: 55 ± 19 years). APACHE-II scores were calculated across all three groups to estimate intensive care unit mortality (Figure 1C). Forty-seven patients (47/77, 61%) experienced mild disease symptoms as indicated by low score values of 0–9. In another 20 (20/77, 26%) and two patients (2/77, 3%), respectively, the clinical condition was rated as moderate and severe, corresponding to score ranges of 10–19 and 20–29, respectively (Figure 1C). Out of those 20 patients with a moderate disease course, 6 (6/35, 17%), 4 (4/11, 36%) and 10 (10/31, 32%) patients belonged to group 1, group 2 and group 3, respectively. As for patients predicted to have a severe course, one patient belonged to each of group 1 and group 2. In eight patients (8/77, 10%), initial assessment according to the APACHE-II scoring system was missed upon admission. In general, the patient population from group 1 was characterized by a mild clinical course (mild: 24/35, 69% vs. moderate: 6/35, 17%), while moderately diseased patients were more common in group 2 (mild: 5/11, 45% vs. moderate: 4/11, 36%) and group 3 (mild: 18/31, 58% vs. moderate: 10/31, 32%), respectively. Only one patient from groups 1 and 2 was graded as critically ill. Within group 1, similar disease severity with only moderate deviations between WON and PC patients were noted (Figure 1D).

Additionally, the disease-specific prognosis was predicted by computing the Glasgow Imrie score at 48 h after admission (Figure 1E). Out of a maximum score of 10 points, 54 patients (54/77, 70%) scored values ≤ 3, while in four patients (4/77, 5%), the score exceeded 3. A Glasgow Imrie score had not been reported in 19 patients (19/77, 25%). The distribution of Glasgow Imrie scores, exemplified by the percentage of patients with mild disease (score ≤ 3), did not vary much among the groups (group 1: 23/35, 66% vs. group 2: 8/11, 73% vs. group 3: 23/31, 73%). Disease severity did not markedly differ in patients with PC and WON within group 1. Compared to the APACHE-II score, fewer patients were classified according to the Glasgow Imrie score, which may explain discrepancies in the percentage numbers of patients assigned to a specific grade of disease severity between both scoring systems.

The median number of endoscopic necrosectomy sessions was two in group 2 (0.5; 2.5) and one in group 3 (0; 3), while there were zero patients with PC from group 1 (0; 0) (Figure 1F). As expected, the majority of necrosectomies were performed in those stent groups exclusively composed of WON patients (group 1: 0 (0; 2) vs. group 2: 2 (1; 3) vs. group 3: 1 (0; 3); Figure 1G).

The median cumulative duration of in-patient care per patient was 38 (16; 54) days (Figure 1H), with PC patients from group 1 (11 (8; 33) days) and WON patients from group 1 (42 (21; 72) days) and group 3 (43 (29; 55) days) accounting for the shortest and longest hospital stays, respectively (Figure 1I). Highly variable lengths of stay were also seen in the PC subgroup and resulted from both patients admitted for elective drainage of symptomatic yet otherwise uncomplicated PC and those hospitalized for emergent drainage, mostly due to infected PC.

Intermediate care (IMC) level medicine was mandated in 41 patients (41/77, 53%) and more likely to be provided to patients from group 2 (6/11, 55%) and group 3 (30/31, 65%) than from group 1 (15/35, 43%), respectively (Figure 1J). However, if group 1 patients were subdivided according to the type of PFC, chances for patients with WON (12/19, 63%) to require transfer to IMC unit were much higher than for patients with PC (3/16, 19%) (Figure 1K).

In total, 11 patients (11/77, 14%) failed to respond to endoscopic drainage and were managed with a surgical step-up approach (group 1: 6/35, 17%) (Figure 1L). The probability to be referred to surgery was higher for patients with WON from group 1 than for patients from group 2 and group 3 (group 1, WON: 5/19, 26% vs. group 2: 1/11, 9% vs. group 3: 4/31, 13%) (Figure 1L,M).

During the observation period, a total of eight patients died in hospital of complications in direct or indirect consequence of their PFC, with no stent group being disproportionately affected (group 1: 4/35, 11% vs. group 2: 2/11, 18% vs. group 3: 2/31, 6%) (Figure 1N,O). Six patients died of septic organ failure related to PFC infection, another two patients died of aspiration pneumonia and lethal arrhythmia, respectively. Since the long-term survival of patients was not actively tracked following their most recent discharge from hospital, the true number of deceased patients may be underestimated.

### 2.2. Procedure- and Disease-Related Complications

**Figure 2:**
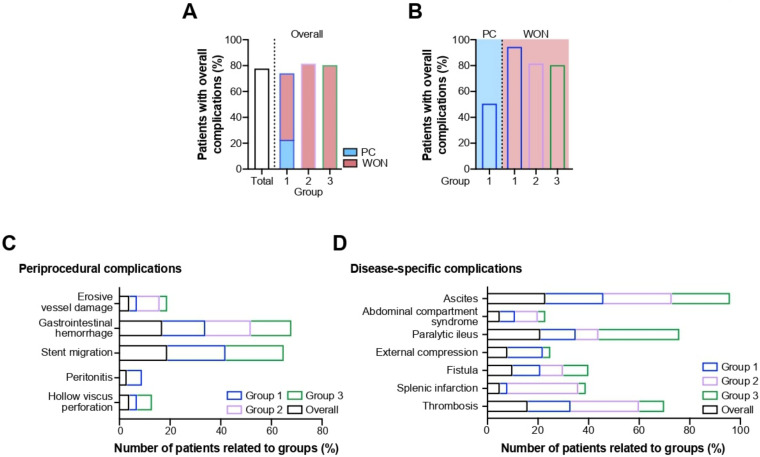
Proportion of patients suffering from any complications related to overall study population and stent group (**A**) and PFC type (**B**). (**C**) Occurrence of periprocedural complications related to overall study population and stent group. (**D**) Occurrence of disease-associated complications related to PFC type.

Sixty patients (60/77, 78%) experienced a complicated disease course, which was related to either the endoscopic intervention, the underlying disease or a combination of both. In general, complications tended to occur more frequently in group 2 and group 3 (9/11, 82% and 25/31, 81%) compared to group 1 (26/35, 74%) (Figure 2A). Analysis of the latter revealed that nearly every patient with WON (18/19, 95%) developed a complication as opposed to only half of all PC patients (8/16, 50%) (Figure 2B). An overview of periprocedural and disease-related adverse events is given in Table 2.

#### 2.2.1. Periprocedural Complications

Subsequent to endoscopic intervention, hollow viscus perforation occurred in only three patients (3/77, 4%; group 1: 1/40, 3%; group 3: 2/31, 6%). Another two patients (2/77, 3%) from group 1, both diagnosed with a WON, developed a peritonitis (group 1: 2/40, 5%) (Figure 2C). Stent migration was noted in a total of 15 patients (15/77, 19%); group 1 vs. group 3 with 8/35, 23% and 7/31, 23%, respectively (Figure 2C). Altogether, 13 patients (13/77, 17%) showed signs of gastrointestinal hemorrhage. The incidence of bleeding was similar across all groups (group 1: 6/35, 17% vs. group 2: 2/11, 18% vs. group 3: 5/31, 16%, respectively) (Figure 2C). Erosive damage to arterial vessels adjacent to the pancreas was confined to one patient from each group, respectively (group 1: 1/35, 3% vs. group 2: 1/11, 9% vs. group 3: 1/31, 3%) (Figure 2C).

#### 2.2.2. Disease-Specific Complications

A total of 12 patients (12/77, 16%) developed a thrombosis of the splenic, portal, and/or mesenteric veins (group 1: 6/35, 17% vs group 2: 3/11, 27% vs. group 3: 3/31, 10%) (Figure 2D). Another four patients (4/77, 5%) were diagnosed with splenic infarction (Figure 2D). Fistulas were found in eight patients (group 1: 4/35, 11% vs. group 2: 1/11, 9% vs. group 3: 3/31, 10%) many of which originated from a disrupted pancreatic duct (*n* = 4) and were communicating with a PFC (*n* = 4) or superior parts of the duodenum (*n* = 1). Less commonly, other sections of the intestine (jejunum, colon) were involved. The diagnosis was usually made visually during endoscopic exploration or by fluoroscopy. External compression of the gastric outlet and/or duodenal loop evolved in six patients (group 1: 5/35, 14% vs. group 2: 0/11, 0% vs. group 3: 1/31, 3%) (Figure 2D). Sixteen patients (16/77, 21%) suffered from a paralytic ileus which became clinically evident in one third of patients in group 3 (group 1: 5/35, 14% vs. group 2: 1/11, 9% vs, group 3: 10/31, 32%) (Figure 2D). In four patients (4/77, 5%) elevated intraabdominal pressures were recorded, meeting the criteria for an abdominal compartment syndrome (group 1: 2/35, 6% vs. group 2: 1/11, 9% vs. group 3: 1/31, 3%) (Figure 2D). Ascites was documented in 18 patients (18/77, 23%) with only marginal inter-group differences (group 1: 8/35, 23% vs. group 2: 3/11, 27% vs. group 3: 7/31, 23%) (Figure 2D).

### 2.3. Infectious Complications

**Figure 3:**
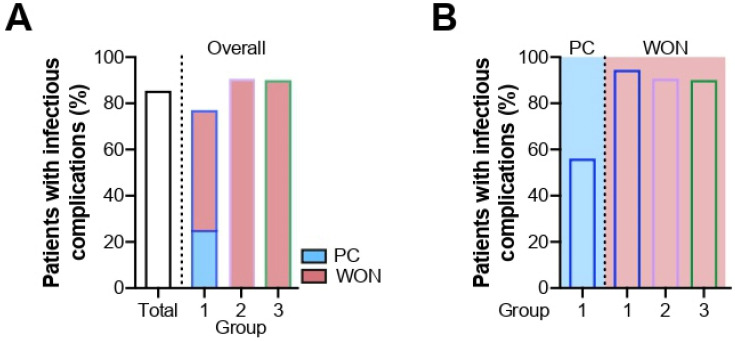
Occurrence of infectious complications related to overall study population and stent group (**A**) and PFC type (**B**).

Hospital-acquired infections occurred in the vast majority of patients (66/77, 86%), demanding effective anti-infective therapy. Confirmed nosocomial infections comprised both general infectious complications and specifically procedure- or disease-associated causes. The overall infection rate was increased in group 2 and group 3 compared to group 1 (group 1: 31/40, 76% vs. group 2: 11/12, 92% vs. group 3: 30/33, 91%) (Figure 3A). While the risk of infection was equally high for all patients with WON regardless of their stent group (WON, group 1: 18/19, 95%, group 2: 10/11, 91%, group 3: 28/31, 90%), it was remarkably lowered in patients with PC (PC, group 1: 9/16, 56%) (Figure 3B). Bacterial and fungal infection rates, as well as the use of anti-infective agents, are outlined in Table 3.

#### 2.3.1. Bacterial Infections

**Figure 4:**
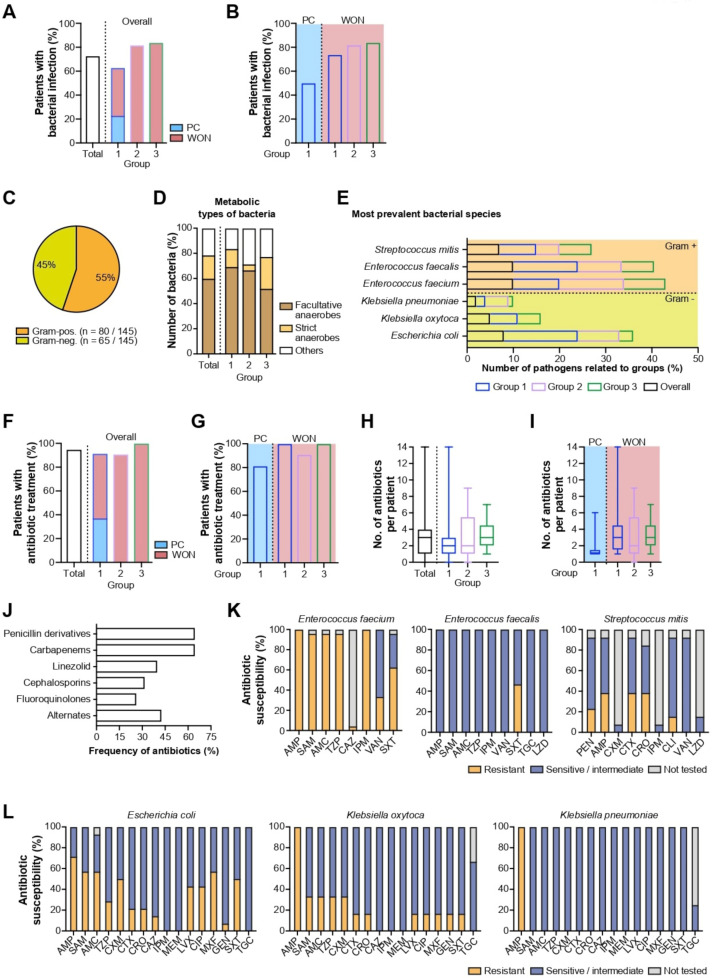
Bacterial infection rates of PFC related to overall study population and stent group (**A**) and PFC type (**B**). (**C**) Classification of bacterial microorganisms based on response to Gram staining. (**D**) Classification of bacterial microorganisms based on metabolic properties. (**E**) Prevalence of the three most common Gram-positive and Gram-negative bacterial species related to overall study population and stent group. Administration rates of antibiotics related to overall study population and stent group (**F**) and PFC type (**G**). Number of antibiotic agents per patient related to overall study population and stent group (**H**) and PFC type (**I**). (**J**) Frequency of antibiotic use per single agent or class. (**K**) Results of antibiotic susceptibility testings for *Enterococcus faecium*, *Enterococcus faecalis*, and *Streptococcus mitis*. (**L**) Results of antibiotic susceptibility testings for *Escherichia coli*, *Klebsiella oxytoca*, and *Klebsiella* *pneumoniae*. Abbreviations: ampicillin (AMP), ampicillin-sulbactam (SAM), amoxicillin-clavulanic acid (AMC), cefotaxime (CTX), cefazolin (CFZ), ceftazidime (CAZ), ceftriaxone (CRO), cefuroxime (CXM), ciprofloxacin (CIP), clindamycin (CLI), gentamicin (GEN), imipenem (IPM), levofloxacin (LVX), linezolid (LZD), meropenem (MEM), moxifloxacin (MXF), penicillin (PEN), piperacillin-tazobactam (TZP), tigecycline (TGC), trimethoprim-sulfamethoxazole (SXT), vancomycin (VAN).

Culture-confirmed bacterial infection of PFC emerged more frequently in patients from group 2 (9/11, 82%) and group 3 (26/31, 84%) compared to group 1 (22/35, 63%) (Figure 4A). Again, in this group, high bacterial infection rates in WON patients were balanced by relatively low values in PC patients (group 1: PC 8/16, 50% vs. WON 14/19, 74%), translating to an overall reduced infection rate. However, a minor difference persisted among WON patients from all groups (Figure 4B).

As illustrated by Figure 4C, Gram staining of specimens from drained collections revealed nearly equal percentages of gram-positive and gram-negative bacteria (80/145, 55% and 65/145, 45%, respectively) across all three groups. In group 1, 49 different bacterial species were identified, while group 2 and group 3 accounted for 21 and 75 species, respectively. In most cases, facultative anaerobe species were identified as the leading pathogen (group 1: 34/49, 69% vs. group 2: 14/21, 67% vs. group 3: 39/75, 52%), while strict anaerobes were of less pathophysiological importance (group 1: 7/49, 14% vs. group 2: 1/21, 5% vs. group 3: 19/75, 25%) (Figure 4D). With regard to gram-negative bacteria, *Escherichia coli* (8/65, 12%), *Klebsiella oxytoca* (5/65, 8%) and *Klebsiella pneumoniae* (2/65, 3%) ranked among the most abundant species, while *Enterococcus faecium* (10/80, 13%), *Enterococcus faecalis* (10/80, 13%) and *Streptococcus mitis* (7/80, 9%) were the three most prevalent Gram-positive species (Figure 4E). A relationship between any of the main three Gram-positive and Gram-negative bacteria and stent type could not be conclusively established owing to small absolute numbers of detected species per stent group. The results of microbiological analysis separated by stent group are depicted in detail in Table 4.

Albeit we observed different rates of bacterial infections among the different stent groups, the frequency of antibiotic treatment did not substantially differ (group 1: 32/35, 91% vs. group 2: 10/11, 91% vs. group 3: 31/31, 100%) (Figure 4F). In group 1, patients with WON were far more likely to be treated with antibiotics than patients with PC (group 1: PC 13/16, 81% vs. WON 19/19, 100%) (Figure 4G).

The median number of antibiotics administered was two in group 1 and group 2 and three in group 3 (Figure 4H). Specifically, group 1 patients with WON received a higher number of antibiotics compared to patients with PC (group 1: PC 1 (1; 1.5) vs. WON 3 (1; 5.5)) (Figure 4I). Penicillin derivatives and carbapenems accounted for approximately two-thirds (47/73, 64% and 47/73, 64%, respectively) of all antibiotic agents used (Figure 4J). Their extensive use is justified by the results of susceptibility testing, confirming that the three most common Gram-negative bacteria species are highly sensitive to the broad-spectrum antibiotic imipenem. Twenty-nine percent of *Escherichia coli* proved to be resistant against piperacillin/tazobactam, while *Klebsiella oxytoca* and *Klebsiella pneumoniae* were 100 percent susceptible. Less promising options include ampicillin, ampicillin/sulbactam, the cephalosporins cefotaxime, cefazolin, ceftazidime, ceftriaxone, and cefuroxime, and the fluoroquinolones levofloxacin, ciprofloxacin and moxifloxacin. (Figure 4K,L). In accordance with that, cephalosporins (23/73, 32%) and fluoroquinolones (19/73, 26%) summed up to less than one-third of all antibiotic agents used (Figure 4J).

Notably, the single agent linezolid was part of antibiotic treatment regimes in 40 percent of cases (29/73, 40%) (Figure 4J). This finding is attributable to the fact that one of the most frequently detected Gram-positive bacterial species, *Enterococcus faecium,* was found to be resistant against vancomycin in exactly one out of three cases (33%) (Figure 4K). By contrast, *Enterococcus faecalis* exhibited susceptibility to all tested antibiotics except for trimethoprim-sulfamethoxazole (50%).

#### 2.3.2. Fungal Infections

**Figure 5:**
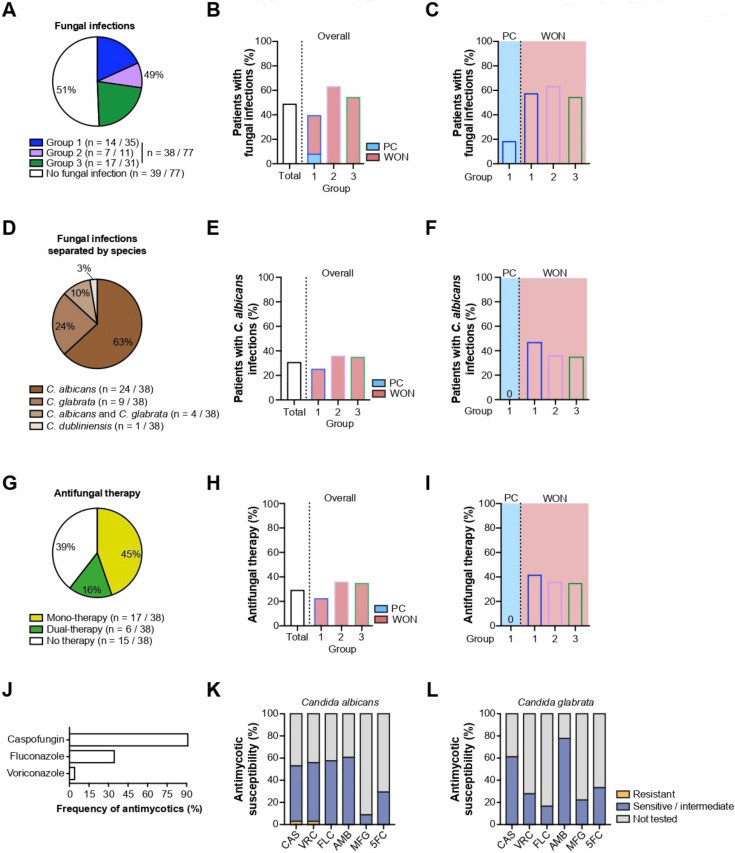
Fungal infection rates of PFC related to overall study population and stent group (**A**,**B**) and PFC type (**C**). (**D**) Percentage distribution of most prevalent *Candida* species. *Candida albicans* infection rates related to overall study population and stent group (**E**) and PFC type (**F**). (**G**) Proportion of patients treated with *n* = 0, *n* = 1, or *n* = 2 antimycotic agents. Administration rates of antimycotics related to overall study population and stent group (**H**) and PFC type (**I**). (**J**) Frequency of antimycotic use per single agent. (**K**) Results of antimycotic susceptibility testings for *Candida albicans*. (**L**) Results of antimycotic susceptibility testings for *Candida glabrata*. Abbreviations: amphotericin B (AMB), caspofungin (CAS), flucytosine (5FC), fluconazole (FLC), micafungin (MFG), voriconazole (VRC).

Next, we focused on the distribution of fungal infections. In nearly half the patient population (38/77, 49%), microbiological sampling of PFC revealed a yeast infection (Figure 5A). Patients from group 2 and group 3 were at a higher risk of contracting a fungal infection compared to group 1 (group 1: 14/35, 40% vs. group 2: 7/11, 64% vs. group 3: 17/31, 55%) (Figure 5B). Again, the overall prevalence in this group was diminished owing to the presence of PC patients who had only a moderate risk of fungal infection, whereas WON patients showed a comparably high level of fungal infections (PC 3/16, 19% vs. WON 11/19, 58%) (Figure 5C).

Among the causative species grown from culture, positive patient samples of *Candida albicans* (24/38, 63%) and *Candida glabrata* (9/38, 24%) were most commonly detected. In rare cases, dual infection with *Candida albicans* and *glabrata* (4/38, 11%) or infection with rare species such as *Candida dubliniensis* (1/38, 3%) were evident (Figure 5D). While nearly half of all group 1 patients with WON contracted a mono-infection with *Candida albicans*, none of the PC patients from the same group were affected (PC 0/16, 0% vs. WON 9/19, 47%). Across all groups, a higher infection rate in WON patients from group 1 was discernible (group 1: 9/19, 47% vs. group 2: 4/11, 36% vs. group 3: 11/31, 35%) (Figure 5E,F).

For most patients with a proven fungal infection, an antifungal monotherapy (17/23, 74%) was sufficient for successful treatment, while the remainder (6/23, 26%) required a second agent (Figure 5G). As anticipated, the administration rates of antifungal therapy were positively correlated with the previously reported heightened risk of fungal infection in group 2 and group 3 compared to group 1 (group 1: 8/35, 23% vs. group 2: 4/11, 36% vs. group 3: 12/31, 39%) (Figure 5H). As before, with the prevalence of fungal infections, the otherwise broad use of antimycotics in WON patients from group 1 contrasted with an administration rate of 0 in PC patients from the same group (PC 0/16, 0% vs. WON 8/19, 42%) (Figure 5I). Caspofungin was by far the most frequently used antimycotic agent, followed by fluconazole and voriconazole (Figure 5J). As opposed to bacterial species, fungi, most of which were identified as *Candida albicans* and *Candida glabrata,* were only sporadically screened for antimycotic resistance. Results of the susceptibility testing revealed that these two *Candida* species are generally sensitive to standard antimycotics, such as the triazole derivatives fluconazole and voriconazole as well as caspofungin and amphotericin B (Figure 5K,L). In only one patient, *Candida albicans* was resistant against caspofungin and voriconazole (Figure 5K). 

## 3. Discussion

Formation of PFC is a serious sequela of acute pancreatitis irrespective of its precipitating factor with a negative impact on morbidity, mortality and duration of reconvalescence. A considerable number of large retrospective studies have investigated the role of LAMS versus DPPS in terms of various relevant outcome variables such as technical success and adverse events [24,25,26]. Our results show that conversion to surgical necrosectomy was more common in WON patients who had exclusively been managed with DPPS compared with LAMS-based approaches. Indeed, a growing body of evidence suggests higher chances of overall clinical benefit in patients treated with metal stents compared to DPPS, with many studies demonstrating a statistically significant effect [27,28,29,30]. A meta-analysis comparing the efficacy of LAMS versus DPPS reported a decreased frequency of pooled adverse events in patients treated with LAMS [31]. Among the reviewed categories of adverse events, net statistical superiority of LAMS was achieved by a significant reduction in hemorrhage [31], which confirmed the results obtained from another previously published meta-analysis [32]. Although no further information on the timeframe of bleeding was provided, it can be presumed that LAMS acts as a plug in the peri-interventional phase by exerting radial forces on adjacent blood vessels, thus decreasing the likelihood of early bleeding events. In our study, no remarkable differences in the incidence of gastrointestinal hemorrhage were observed among all stent groups. On the other hand, in a randomized prospective trial comparing the performance of LAMS versus DPPS with regard to resolution of WON, Bang et al. emphasized the high number of delayed gastrointestinal hemorrhages in patients with indwelling LAMS, hypothesizing that, due to friction between the stent and vasculature of the collapsed necrotic cavity, vascular damage with subsequent bleeding is induced [33]. In response to growing expertise in our own and other endoscopic facilities, the standard practice has been amended to include an additional DPPS as a spacer to prevent erosive bleeding caused by the wire surface of LAMS [34].

No matter which device for drainage is chosen, perforation can be regarded as a rare adverse event if the PFC is tapped under continuous EUS surveillance. In conjunction with this safety precaution, DPPS were associated with similar perforation rates compared to LAMS but entailed a higher risk if non-EUS-guided cases were analyzed as well [35]. In our study, needle puncture and insertion of the guidewire for subsequent stent positioning were exclusively achieved by EUS. In accordance with the previously cited findings, the incidence of perforations in our study was minute, affecting only one patient from group 1 (2.9%) and two patients from group 3 (6.5%). These data imply that patients with DPPS are more likely to incur a perforation, in particular when compared with LAMS only (0%). These results are somewhat contradictory to another clinical study, suggesting a higher risk of perforation in patients in whom access to the necrotic cavity was created via LAMS (4%) as opposed to patients with DPPS (1%) [36]. Notably, the authors admitted to preceding unsuccessful attempts of stent deployment in all three cases of perforation in the LAMS group. In light of the low absolute numbers of perforation, it is questionable whether they permit robust conclusions about a stent type-dependent risk and whether they may, to some extent, be ascribed to the individual endoscopists’ skills.

Stent migration is another adverse event characteristic of transmural endoscopic drainage of PFC. Additional positional stability can be gained by introducing LAMS, equipped with a saddle shape and anchoring flanges [37,38]. In a large international multi-center study, migration occurred more frequently in patients with DPPS (7%) compared to LAMS (3%) [39]. Not surprisingly, in our study, stent migration was exclusively registered in patients with DPPS as part of their endoscopic management.

To date, there is still a paucity of data available on the microbial spectrum and its clinical relevance in infected pancreatic WON. A retrospective review on 78 patients who underwent endoscopic transmural drainage with necrosectomies suggests an intimate association between surrogate parameters of critical disease, such as the need for ICU monitoring and infected pancreatic necrosis [40]. Most commonly, Gram-positive *Enterococcus* species (45%) and Gram-negative Enterobacteriaceae (42%) were detected upon cultivation of PFC secretions [40]. In our study, preliminary Gram staining of specimens from drained collections was characterized by a similar ratio of Gram-positive:Gram-negative microorganisms. Furthermore, our dataset displayed a gradual increase in the prevalence of bacterial infection, being most pronounced in patients fitted with a LAMS (group 2 and group 3) compared to DPPS only (group 1). This development was paralleled by prolonged in-hospital stays and higher probability of IMC transfer in these groups. These results indicate that bacterial infection of PFC frequently coincides with LAMS-mediated drainage and appears to be correlated with more protracted and complicated disease courses.

Fungal infection of necrotic areas represents another aggravating factor of necrotizing pancreatitis [41]. Currently, few studies are dedicated to determining its influence on the clinical course. In several single-center retrospective studies in patients undergoing open surgery for pancreatic necrosis, colonization of the resectate with *Candida* ranged from 17 to 35% [42,43]. Within an 8-year observation period, the cumulative detection rate of fungi in patients with WON treated with endoscopic drainage and necrosectomy rose to 46% compared to 16% upon index endoscopy. Unlike a locally confined infection, concomitant fungal dissemination into the peripheral bloodstream was found to significantly deteriorate overall survival [44]. To our best knowledge, there are no current studies evaluating the impact of diverse stent models on the general proneness of patients with necrotizing pancreatitis to fungal infection. Furthermore, for the first time, the isolation of distinct Candida species from clinical specimens has been contextually linked with the stent type used. Again, our study revealed that patients whose interventional strategy included a LAMS (group 2 and group 3) were more frequently affected by fungal complications compared to patients with DPPS only (group 1). Results from classification into different *Candida* strains were in accordance with previously published literature, with *Candida albicans* being the most abundant yeast species [45]. We were able to demonstrate that the risk of infection with this fungus was markedly increased in patients with a WON compared to PC.

At first sight, our findings ostensibly support the assumption that endoscopic drainage by means of a metal stent such as LAMS is associated with an increased risk of bacterial and fungal infections, prolonged hospitalizations and extensive use of anti-infective agents. Conceivably, metal stents with their large lumen diameter may favor translocation of both bacterial and fungal flora into the drained cavity and, hence, facilitate infection of its contents. However, splitting up the heterogeneous population of group 1 according to the type of PFC unveiled that the risk of infectious complications in WON patients remained unaltered across all stent groups. This pattern recurred when indirect indicators of disease severity such as IMC transfer or length of hospital stay were analyzed, suggesting that the risk of infection is chiefly determined by the therapeutic complexity of the underlying PFC rather than the stent type itself. Due to inconsistent time points of collecting specimens and follow-up sampling not being routinely intended, it still remains uncertain as to whether infection is mainly the consequence of passive carry-over through the stent lumen or whether scheduled necrosectomies or comparably invasive endoscopic measures may additionally promote colonization of the PFC. Furthermore, a suppressed immune response, which is thought to counterbalance the initially excessive systemic inflammation in patients with acute pancreatitis, may increase susceptibility to infections in the later course [46].

## 4. Materials and Methods

### 4.1. Study Design and Data Acquisition

The electronic medical database at the university hospital Ulm was systematically screened for patients with transgastric endoscopic drainage of post-pancreatitic PC or WON with a self-expanding or a non-expanding stent. A preliminary query yielded 91 patients who had undergone one of the procedures indicated above in our department during the time period of 2014–2020. For patients to be eligible for inclusion into the study, only the following two criteria needed to be fulfilled: age older than 18 years and exclusion of pregnancy in female patients of child-bearing age. Reports on identical procedures conducted in external health care facilities were automatically entered into the patients’ file and incorporated into the analysis. A fraction (*n* = 32) of this cohort was evaluated under a different scope and topic in an independent study [47]. The final study population encompassed 77 individuals who were considered suitable for enrollment in this study (Figure 1A).

The definitions of PC and WON, respectively, were adopted from the revised Atlanta classification. The technical feasibility of endoscopic drainage was determined after at least 7–10 days after the onset of clinical symptoms by cross-sectional imaging techniques such as computed tomography or magnetic resonance imaging to localize the PFC and gauge their minimum distance to the gastric wall. At the same time, the initial diagnosis was ascertained to exclude cystic pancreatic neoplasms, pseudo-aneurysms or other non-inflammatory mass lesions. Prior to any intervention, generally accepted cut-off values for coagulation (international normalized ratio < 1.5) and thrombocytes (>50 /nL) were applied. In each patient, correct stent position and drainage function was verified immediately upon completion of the endoscopic procedure. In patients with suspicion of disconnected pancreatic duct syndrome, magnetic resonance cholangiopancreatography or endosonography were regularly used to examine the major pancreatic duct before definitive stent removal.

Compelling indications for endoscopic drainage of PC and WON included the following: (1) confirmed or suspected infection with or without multiple organ failure and systemic inflammation; (2) absence of spontaneous regression or continuous enlargement with compression of the common bile duct and/or gastric outlet/duodenal loop resulting in intractable pain, cholestasis, recurrent nausea/vomiting and compromised enteric nutrition; (3) relief of abdominal compartment syndrome.

### 4.2. Stent Devices and Description of Procedure

In all patients, stent placement was performed with a dual imaging approach using both linear array EUS and fluoroscopy. PFC was localized by EUS under flow guidance for prevention of vascular injury. For stent placement, we used different types of LAMS, either the Hot Axios Stent system (Boston Scientific, Marlborough, MA, USA, stent deployment as previously described [48]) or biflanched HANAROSTENT PseudoCyst fully covered metal stents (M.I. Tech, Seoul, Korea). For HANAROSTENT and DPPS application, we used a sequential deployment procedure: the cavity was accessed by a 19 Gauge Needle (Cook Medical, Winston-Salem, NC, USA), correct position was confirmed by aspiration of secretions and X-ray fluoroscopy for subsequent guidewire insertion (0.035 inch). Samples for microbial cultivation were usually obtained by aspiration at this point. After preparation with a ring knife (MTW-Endoskopie Manufaktur, Model Prof. Dr. U. Will, Wesel, Germany), a dilatation catheter (sizes 5/7/10 French) was used to prepare stent placement. Routinely, standard DPPS (10 French) of different lengths (total length range 60–160 mm) or HANAROSTENT Metal Plumberstents (either 20 or 40 mm total length) were used. A decision regarding the choice of stent was made depending on the EUS-based fluid texture (DPPS: predominantly liquid collections; LAMS: predominantly solid collections; LAMS + DPPS: mixed composition; prophylaxis of delayed bleeding).

### 4.3. Rationale for Anti-Infective Management

Antibiotic treatment was usually initiated with therapeutic intent upon suspecting a bacterial or fungal infection. Antibiotic prophylaxis is generally not used in our center according to (inter-) national guidelines [20,21]. However, we cannot exclude that patients referred to our center had previously received prophylactic antibiotics due to clinical deterioration upon admission to the referring hospital. At our center, the decision to administer antibiotics was based on common clinical signs of infection such as fever, chills, hemodynamic instability, organ dysfunction and laboratory markers of inflammation, e.g., a significant rise in leukocyte counts and C-reactive protein. In case no antibiotic sensitivity tests were available, the choice of antibiotic regime followed in-house guidelines for empiric antimicrobial therapy. If the later course sampling of PFC revealed one or several infectious agents, antibiotic therapy was adjusted according to the antibiogram—this could mean that the current regime was switched to a more effective antibiotic or augmented by another antibiotic agent to target a specific bacterial pathogen. If fungus species were detected, the anti-infective coverage was extended to include an antimycotic agent. Issues of tissue penetration, oral availability and side effects relevant to a patient’s condition, e.g., hematological disorders, nephrotoxicity, etc., were individually considered and discussed with microbiologists.

### 4.4. Outcome Measures

Besides the primary endpoints “Total duration of hospital stay per patient” and “in-hospital mortality”, a principal aspect of this study was the prevalence of complications arising at any time point during the period under review. A complication was defined as an unfavorable or unintended event, temporally associated with either the disease or the intervention itself, or a combination of both. This ample endpoint was divided into typically procedure-related adverse events such as bleeding, perforation and stent migration, and the entirety of bacterial and fungal infections confirmed by positive microbiological cultures from drained collections. The remainder of the complications sorted by organ systems were listed but not described in detail. Administration of antibiotic or antifungal therapy, assessment of disease severity, duration of hospital stay, transfer to IMC unit and necessity of surgical debridement were considered secondary endpoints.

### 4.5. Statistical Analysis

The present study is a retrospective cohort analysis. Patients who had undergone endoscopic drainage of a PFC were divided into 3 groups based on the stent type used: DPPS only (group 1); LAMS only (group 2); and a combination of DPPS and LAMS (group 3). With the exception of group 1, comprising both PC and WON, the remaining two groups included only patients with WON. Where applicable, data were reported separately per subgroup.

Owing to the retrospective study design and limited sample size, we chose an exclusively descriptive approach. Besides the absolute numbers, nominal variables were expressed as percentages and their respective confidence intervals (CI). Scalable variables were represented as median values and their respective 25th and 75th percentiles. In addition, the number of necrosectomies and total duration of hospital stays per patient were plotted with their maximum and minimum values.

## 5. Conclusions

This retrospective study was designed to pursue the question of whether stents fabricated from metal or plastic differently affect general outcome parameters and adverse events inherent in endoscopic drainage of PFC. A major objective of this study was to interrogate whether the choice of stent influences the risk of bacterial and fungal infection. Based on the analyzed data, it can be stated that patients with WON compared to PC seem to be predisposed to a more complicated disease course, manifesting itself in a higher prevalence of bacterial and fungal infections of the necrotic cavity, prolonged in-hospital stays and increased need of intermediate care monitoring. The type of stent does not seem to interfere with these parameters. However, the retrospective study design prohibits speculations on a causal relationship between the observed findings. Another limiting factor concerns the heterogenous structure of the patient population for which no stringent inclusion or exclusion criteria have been defined. Furthermore, it must be considered that statistical validity is substantially reduced due to a limited patient sample size. Intriguingly, general and disease-specific prognostic scores reported upon admission did not reflect the differences between WON and PC patients that had retrospectively been observed for a variety of outcome parameters. However, despite several shortcomings, this study may provide valuable incentives to debate on a presumptive connection between different stent types and the risk of microbial infection.

Therefore, in future prospective cohort studies, larger sample sizes are warranted to further clarify the important aspects addressed in this study and to serve as potential guidance for the selection of anti-infective agents grounded on empirical evidence.

## Figures and Tables

**Table 1:** Overview of the study population, including primary and secondary clinical endpoints.

	Overall	Group 1	PC	WON	Group 2	Group 3
Number of Patients	77	35	16	19	11	31
Female, *n* (%)	23 (30)	10 (29)	3 (19)	7 (37)	4 (36)	9 (29)
Male, *n* (%)	54 (70)	25 (71)	13 (81)	12 (63)	7 (64)	22 (71)
Age (years) (mean ± standard deviation)	55 ± 17	55 ± 14	49 ± 14	59 ± 12	56 ± 17	55 ± 19
Apache-II score						
0–9, *n* (%)	47 (61)	24 (69)	12 (75)	12 (63)	5 (45)	18 (58)
10–19, *n* (%)	20 (26)	6 (17)	3 (19)	3 (16)	4 (36)	10 (32)
20–29, *n* (%)	2 (3)	1 (3)	0 (0)	1 (5)	1 (9)	0 (0)
>30, *n* (%)	0 (0)	0 (0)	0 (0)	0 (0)	0 (0)	0 (0)
Glasgow Imrie score						
≤3, *n* (%)	54 (70)	23 (66)	9 (56)	14 (74)	8 (73)	8 (73)
>3, *n* (%)	4 (5)	2 (6)	1 (6)	1 (5)	0 (0)	0 (0)
not recorded, *n* (%)	19 (25)	10 (29)	6 (38)	4 (21)	3 (27)	3 (27)
Total duration of hospital stay per patient (days) (median, 25%; 75% quartile)	38 (16; 54)	32 (11; 56)	11 (7.75; 33)	42 (21; 72)	30 (14.5; 43)	43 (29; 55)
Number of endoscopic necrosectomy sessions (median, 25%; 75% quartile)	0 (0; 2)	0 (0; 0)	0 (0; 0)	0 (0; 0)	2 (1; 2.75)	1 (0; 3)
Conversion to surgery, *n* (%; 95% CI)	11 (14; 8–24%)	6 (17; 8–33%)	1 (6; 0–30%)	5 (26; 11–49%)	1 (9; 0–40%)	4 (13; 5–29%)
Transfer to IMC, *n* (%; CI)	41 (53, 42–64%)	15 (43; 28–59%)	3 (19; 6–44%)	12 (63; 41–81%)	6 (55; 28–79%)	20 (65; 47–79%)
Mortality, *n* (%, 95% CI)	8 (10; 5–19%)	4 (11; 4–27%)	2 (13; 2–37%)	2 (11; 2–33%)	2 (18; 4–49%)	2 (6; 7–22%)

**Table 2:** Periprocedural and disease-related adverse events.

	Overall	Group 1	PC	WON	Group 2	Group 3
**Overall complications**	60 (78; 67–86%)	26 (74; 58–86%)	8 (50; 28–72%)	18 (95; 74–100%)	9 (82; 51–96%)	25 (81; 63–91%)
**Periprocedural adverse events**						
*Gastrointestinal hemorrhage, n* (%; 95% CI)	13 (17; 10–27%)	6 (17; 8–33%)	2 (13; 2–37%)	4 (21; 8–44%)	2 (18; 4–49%)	5 (16; 7–33%)
*Perforation, n* (%; 95% CI)	3 (4, 1–11%)	1 (3; 0–16%)	0 (0; 0–23%)	1 (5; 0–26%)	0 (0; 0–30%)	2 (6; 1–22%)
*Stent migration, n* (%; 95% CI)	15 (19, 12–30%)	8 (23; 12–39%)	2 (13; 2–37%)	6 (32; 15–54%)	0 (0; 0–30%)	7 (23; 11–40%)
*Erosive vessel damage, n* (%)	3 (4)	1 (3)	0 (0)	1 (5)	1 (9)	1 (3)
**Cardiovascular complications**						
*Hemodynamic instability, n* (%)	2 (3)	0 (0)	0 (0)	0 (0)	1 (9)	1 (3)
*Thrombosis, n* (%)	12 (16)	6 (17)	3 (19)	3 (16)	3 (27)	3 (10)
*Splenic infarction, n* (%)	4 (5)	1 (3)	1 (6)	0 (0)	2 (28)	1 (3)
**Respiratory complications**						
*Pneumonia, n* (%)	17 (22)	7 (20)	3 (19)	4 (21)	5 (45)	5 (16)
*Respiratory failure, n* (%)	5 (6)	2 (6)	1 (6)	1 (5)	1 (9)	2 (6)
*Need for mechanical ventilation, n* (%)	13 (17)	5 (14)	2 (13)	3 (16)	4 (36)	4 (13)
*Pleural effusion, n* (%)	32 (42)	14 (40)	1 (6)	13 (68)	5 (45)	13 (42)
**Gastrointestinal complications**						
*Fistula, n* (%)	8 (10)	4 (11)	1 (6)	3 (16)	1 (9)	3 (10)
*Ascites, n* (%)	18 (23)	8 (23)	3 (19)	5 (26)	3 (27)	7 (23)
*Ileus, n* (%)	16 (21)	5 (14)	1 (6)	4 (21)	1 (9)	10 (32)
*Abdominal compartment syndrome, n* (%)	4 (5)	2 (6)	0 (0)	2 (11)	1 (9)	1 (3)
*Gastric/duodenal outlet syndrome, n* (%)	6 (8)	5 (14)	3 (19)	2 (11)	0 (0)	1 (3)
*Peritonitis, n* (%; CI)	2 (3, 0–10%)	2 (6; 1–20%)	0 (0; 0–23%)	2 (11; 2–33%)	0 (0; 0–30%)	0 (0; 0–13%)
*Biliary complications, n* (%)	6 (8)	4 (11)	2 (13)	2 (11)	1 (9)	1 (3)
**Renal complications**						
*Acute renal failure, n* (%)	18 (23)	5 (14)	2 (13)	3 (16)	5 (45)	8 (26)

**Table 3:** Bacterial and fungal infections and anti-infective use.

	Overall	Group 1	PC	WON	Group 2	Group 3
**Overall infection, (%; 95% CI)**	66 (86, 76–92%)	28 (80; 64–90%)	9 (56; 33–77%)	18 (95; 74–100%)	10 (91; 60–100%)	28 (90; 74–97%)
**Bacterial infection, *n* (%; 95% CI)**	56 (73; 62–81%)	22 (63; 46–77%)	8 (50; 28–72%)	14 (74; 51–89%)	9 (82; 51–96%)	26 (84; 67–93%)
*Bacterial pathogens, n* (%)	145 (100)	49 (100)	11 (100)	38 (100)	21 (100)	75 (100)
*Gram-positive, n* (%; CI)	80 (55; 47–63%)	26 (53; 39–66%)	6 (55; 28–79%)	20 (53; 37–68%)	11 (52; 32–72%)	43 (57; 46–68%)
*Gram-negative, n* (%; CI)	65 (45; 37–53%)	23 (47; 34–61%)	5 (45; 21–72%)	18 (47; 32–63%)	10 (48; 28–68%)	32 (43; 32–54%)
*Strict anaerobes, n* (%)	27 (19)	7 (14)	4 (36)	5 (8)	1 (5)	19 (25)
*Facultative anaerobes, n* (%)	87 (60)	34 (69)	6 (55)	28 (74)	14 (67)	39 (52)
Use of antibiotics, *n* (%; CI)	73 (95, 87–98%)	32 (91; 77–98%)	13 (81; 56–94%)	19 (100; 80–100%)	10 (91; 60–100%)	31 (100; 87–100%)
Number of antibiotics per patient (median, 25%; 75% quartile)	3 (1; 4)	2 (1; 3)	1 (1; 1.5)	3 (1.5; 4.5)	2 (1; 5.5)	3 (2; 4.5)
Fluoroquinolones	19					
Cephalosporines	23					
Penicillins and β-lactamase inhibitors	47					
Carbapenems	47					
Linezolide	29					
Others	31					
**Fungal infection, *n* (%; 95% CI)**	38 (49; 38–60%)	14 (40; 26–56%)	3 (19; 6–44%)	11 (58; 36–77%)	7 (64; 35–85%)	17 (55; 38–71%)
*Candida albicans, n* (%, 95% CI)	24 (31; 22–42%)	9 (26; 14–42%)	0 (0; 0–23%)	9 (47; 27–68%)	4 (36; 15–65%)	11 (35; 21–53%)
*Candida glabrata, n* (%, 95% CI)	9 (12; 6–21)	4 (11; 4–27%)	2 (13; 2–37%)	2 (11; 2–33%)	2 (18; 4–49%)	3 (10; 3–26%)
*Candida dubliniensis, n* (%, 95% CI)	1 (1)	0 (0)	0 (0)	0 (0)	1 (9; 0–40%)	0 (0)
*Candida tropicalis + albicans + glabrata, n* (%)	0 (0)	0 (0)	0 (0)	0 (0)	0 (0)	0 (0)
Use of antimycotis, *n* (%; 95% CI)	23 (31)	8 (23;12–39%)	0 (0; 0–23%)	8 (42; 23–63%)	4 (36; 15–65%)	11 (36; 19–55%)
0, *n* (%)	54 (70)	27 (77)	16 (100)	11 (58)	7 (64)	20 (65)
1, *n* (%)	17 (22)	5 (14)	0 (0)	5 (26)	2 (18)	10 (32)
2, *n* (%)	6 (8)	3 (9)	0 (0)	3 (16)	2 (18)	1 (3)
Voriconazole	1					
Fluconazole	8					
Caspofungin	21					

**Table 4:** Complete spectrum of bacterial species detected in PFC specimens.

	Overall	Group 1	Group 2	Group 3
Gram-positive bacteria	80	26	11	43
Facultative anaerobes	54	19	9	26
*Enterococcus faecium*	15	5	3	7
*Enterococcus faecalis*	14	7	2	5
*Streptococcus mitis*	10	4	1	5
*Staphylococcus haemolyticus*	5	1	1	3
*Staphylococcus hominis*	1	0	0	1
*Staphylococcus aureus*	2	1	1	0
*Staphylococcus epidermidis*	4	1	0	3
*Streptococcus oralis*	1	0	0	1
*Streptococcus salivarius*	1	0	0	1
*Streptococcus sanguinis*	1	0	1	0
Strict Anaerobes	7	3	0	4
*Lactobacillus* species	3	2	0	1
*Actinomyces* species	1	0	0	1
*Bifidobacterium* species	1	0	0	1
*Peptoniphilus saccharolyticus*	1	0	0	1
*Propionibacterium* acnes	1	1	0	0
Others	19	4	2	13
*Streptococcus anginosus*	9	1	1	7
*Corynebacterium* species	1	0	0	1
*Streptococcus* species	4	2	0	2
Other Gram-positive species	5	1	1	3
Gram-negative bacteria	65	23	10	32
Facultative Anaerobes	33	15	5	13
*Escherichia coli*	12	8	2	2
*Klebsiella oxytoca*	7	3	0	4
*Klebsiella pneumoniae*	3	1	1	1
*Enterobacter cloacae*	4	0	1	3
*Citrobacter freundii*	2	0	1	1
*Proteus vulgaris*	1	0	0	1
*Aeromonas caviae*	1	1	0	0
*Eikenella* species	1	0	0	1
*Hafnia alvei*	1	1	0	0
*Morganella morganii*	1	1	0	0
Strict Anaerobes	20	4	1	15
*Bacteroides fragilis*	3	1	1	1
*Prevotella buccae*	3	0	0	3
*Veillonella* species	3	1	0	2
*Prevotella denticola*	2	0	0	2
*Prevotella oralis*	2	0	0	2
*Prevotella disiens*	1	0	0	1
*Prevotella melaninogenica*	1	1	0	0
*Prevotella* species	3	1	0	2
*Megasphaera* species	1	0	0	1
*Fusobacterium* species	1	0	0	1
Others	12	4	4	4
*Pseudomonas aeruginosa*	3	1	1	1
*Acinetobacter Iwolfii*	1	0	0	1
*Stenotrophomonas maltophilia*	2	1	1	0
Other Gram-negative species	6	2	2	2

## Data Availability

Not applicable.

## References

[1] Popa C.C., Badiu D.C., Rusu O.C., Grigorean V.T., Neagu S.I., Strugaru C.R. (2016). Mortality prognostic factors in acute pancreatitis. J. Med. Life.

[2] Shi N., Liu T., de la Iglesia-Garcia D., Deng L., Jin T., Lan L., Zhu P., Hu W., Zhou Z., Singh V. (2020). Duration of organ failure impacts mortality in acute pancreatitis. Gut.

[3] Ouyang G., Pan G., Liu Q., Wu Y., Liu Z., Lu W., Li S., Zhou Z., Wen Y. (2020). The global, regional, and national burden of pancreatitis in 195 countries and territories, 1990–2017: A systematic analysis for the Global Burden of Disease Study 2017. BMC Med..

[4] Dumnicka P., Maduzia D., Ceranowicz P., Olszanecki R., Drożdż R., Kuśnierz-Cabala B. (2017). The Interplay between Inflammation, Coagulation and Endothelial Injury in the Early Phase of Acute Pancreatitis: Clinical Implications. Int. J. Mol. Sci..

[5] Komara N.L., Paragomi P., Greer P.J., Wilson A.S., Breze C., Papachristou G.I., Whitcomb D.C. (2020). Severe acute pancreatitis: Capillary permeability model linking systemic inflammation to multiorgan failure. Am. J. Physiol. Gastrointest. Liver Physiol..

[6] Banks P.A., Bollen T.L., Dervenis C., Gooszen H.G., Johnson C.D., Sarr M.G., Tsiotos G.G., Vege S.S. (2013). Classification of acute pancreatitis—2012: Revision of the Atlanta classification and definitions by international consensus. Gut.

[7] Grimm H., Binmoeller K.F., Soehendra N. (1992). Endosonography-guided drainage of a pancreatic pseudocyst. Gastrointest. Endosc..

[8] Wiersema M.J. (1996). Endosonography-guided cystoduodenostomy with a therapeutic ultrasound endoscope. Gastrointest. Endosc..

[9] Kawakami H., Itoi T., Sakamoto N. (2014). Endoscopic ultrasound-guided transluminal drainage for peripancreatic fluid collections: Where are we now?. Gut Liver.

[10] Li J., Zhang Q., Zhou A., Zhao G., Li P. (2021). Comparative outcomes of endoscopic ultrasound-guided lumen-apposing mental stents drainage for pancreatic pseudocysts and walled-off necrosis: Case series and meta-analysis. Chronic Dis. Transl. Med..

[11] Stecher S.S., Simon P., Friesecke S., Glitsch A., Kühn J.P., Lerch M.M., Mayerle J. (2017). Delayed severe bleeding complications after treatment of pancreatic fluid collections with lumen-apposing metal stents. Gut.

[12] Beger H.G., Bittner R., Block S., Büchler M. (1986). Bacterial contamination of pancreatic necrosis. A prospective clinical study. Gastroenterology.

[13] Gerzof S.G., Banks P.A., Robbins A.H., Johnson W.C., Spechler S.J., Wetzner S.M., Snider J.M., Langevin R.E., Jay M.E. (1987). Early diagnosis of pancreatic infection by computed tomography-guided aspiration. Gastroenterology.

[14] Beger H.G., Büchler M., Bittner R., Block S., Nevalainen T., Roscher R. (1988). Necrosectomy and postoperative local lavage in necrotizing pancreatitis. Br. J. Surg..

[15] Delcenserie R., Yzet T., Ducroix J.P. (1996). Prophylactic antibiotics in treatment of severe acute alcoholic pancreatitis. Pancreas.

[16] Ignatavicius P., Vitkauskiene A., Pundzius J., Dambrauskas Z., Barauskas G. (2012). Effects of prophylactic antibiotics in acute pancreatitis. HPB Off. J. Int. Hepato Pancreato Biliary Assoc..

[17] Pederzoli P., Bassi C., Vesentini S., Campedelli A. (1993). A randomized multicenter clinical trial of antibiotic prophylaxis of septic complications in acute necrotizing pancreatitis with imipenem. Surg. Gynecol. Obstet..

[18] Schwarz M., Isenmann R., Meyer H., Beger H.G. (1997). Antibiotic use in necrotizing pancreatitis. Results of a controlled study. Dtsch. Med. Wochenschr..

[19] Villatoro E., Mulla M., Larvin M. (2010). Antibiotic therapy for prophylaxis against infection of pancreatic necrosis in acute pancreatitis. Cochrane Database Syst. Rev..

[20] (2013). IAP/APA evidence-based guidelines for the management of acute pancreatitis. Pancreatol. Off. J. Int. Assoc. Pancreatol. IAP.

[21] Beyer G.H., Michl P., Gress T.M., Algül H., Neesse A., Meining A., Seufferlein T.W., Rosendahl J., Kahl S., Keller J. (2021). S3-Leitlinie Pankreatitis. Leitlinie der Deutschen Gesellschaft für Gastroenterologie, Verdauungs- und Stoffwechselkrankheiten (DGVS). Z. Gastroenterol..

[22] Singh R.R., Mitchell W., David Y., Cheesman A., Dixon R.E., Nagula S., DiMaio C.J., Greenwald D.A., Kumta N.A. (2021). Pancreatic Fungal Infection in Patients With Necrotizing Pancreatitis: A Systematic Review and Meta-analysis. J. Clin. Gastroenterol..

[23] Trikudanathan G., Navaneethan U., Vege S.S. (2011). Intra-abdominal fungal infections complicating acute pancreatitis: A review. Am. J. Gastroenterol..

[24] Boxhoorn L., Voermans R.P., Bouwense S.A., Bruno M.J., Verdonk R.C., Boermeester M.A., van Santvoort H.C., Besselink M.G. (2020). Acute pancreatitis. Lancet.

[25] Karsenti D., Bourlier P., Dorval E., Scotto B., Giraudeau B., Lanotte R., de Calan L., Mesny J., Lagarrigue F., Metman E. (2002). Morbidity and mortality of acute pancreatitis. Prospective study in a French university hospital. Presse Med..

[26] Yadav J., Yadav S.K., Kumar S., Baxla R.G., Sinha D.K., Bodra P., Besra R.C., Baski B.M., Prakash O., Anand A. (2016). Predicting morbidity and mortality in acute pancreatitis in an Indian population: A comparative study of the BISAP score, Ranson’s score and CT severity index. Gastroenterol. Rep..

[27] Mohan B.P., Jayaraj M., Asokkumar R., Shakhatreh M., Pahal P., Ponnada S., Navaneethan U., Adler D.G. (2019). Lumen apposing metal stents in drainage of pancreatic walled-off necrosis, are they any better than plastic stents? A systematic review and meta-analysis of studies published since the revised Atlanta classification of pancreatic fluid collections. Endosc. Ultrasound.

[28] Shah R.J., Shah J.N., Waxman I., Kowalski T.E., Sanchez-Yague A., Nieto J., Brauer B.C., Gaidhane M., Kahaleh M. (2015). Safety and efficacy of endoscopic ultrasound-guided drainage of pancreatic fluid collections with lumen-apposing covered self-expanding metal stents. Clin. Gastroenterol. Hepatol. Off. Clin. Pract. J. Am. Gastroenterol. Assoc..

[29] Walter D., Will U., Sanchez-Yague A., Brenke D., Hampe J., Wollny H., López-Jamar J.M., Jechart G., Vilmann P., Gornals J.B. (2015). A novel lumen-apposing metal stent for endoscopic ultrasound-guided drainage of pancreatic fluid collections: A prospective cohort study. Endoscopy.

[30] Yoon S.B., Lee I.S., Choi M.G. (2018). Metal versus plastic stents for drainage of pancreatic fluid collection: A meta-analysis. United Eur. Gastroenterol. J..

[31] Tan S., Zhong C., Ren Y., Luo X., Xu J., Peng Y., Fu X., Tang X. (2020). Are Lumen-Apposing Metal Stents More Effective Than Plastic Stents for the Management of Pancreatic Fluid Collections: An Updated Systematic Review and Meta-analysis. Gastroenterol. Res. Pract..

[32] Bazerbachi F., Sawas T., Vargas E.J., Prokop L.J., Chari S.T., Gleeson F.C., Levy M.J., Martin J., Petersen B.T., Pearson R.K. (2018). Metal stents versus plastic stents for the management of pancreatic walled-off necrosis: A systematic review and meta-analysis. Gastrointest. Endosc..

[33] Bang J.Y., Navaneethan U., Hasan M.K., Sutton B., Hawes R., Varadarajulu S. (2019). Non-superiority of lumen-apposing metal stents over plastic stents for drainage of walled-off necrosis in a randomised trial. Gut.

[34] Ge P.S., Young J.Y., Jirapinyo P., Dong W., Ryou M., Thompson C.C. (2020). Comparative Study Evaluating Lumen Apposing Metal Stents Versus Double Pigtail Plastic Stents for Treatment of Walled-Off Necrosis. Pancreas.

[35] Chandrasekhara V., Barthet M., Devière J., Bazerbachi F., Lakhtakia S., Easler J.J., Peetermans J.A., McMullen E., Gjata O., Gourlay M.L. (2020). Safety and efficacy of lumen-apposing metal stents versus plastic stents to treat walled-off pancreatic necrosis: Systematic review and meta-analysis. Endosc. Int. Open.

[36] Siddiqui A.A., Kowalski T.E., Loren D.E., Khalid A., Soomro A., Mazhar S.M., Isby L., Kahaleh M., Karia K., Yoo J. (2017). Fully covered self-expanding metal stents versus lumen-apposing fully covered self-expanding metal stent versus plastic stents for endoscopic drainage of pancreatic walled-off necrosis: Clinical outcomes and success. Gastrointest. Endosc..

[37] Boxhoorn L., Fockens P., Besselink M.G., Bruno M.J., van Hooft J.E., Verdonk R.C., Voermans R.P. (2018). Endoscopic Management of Infected Necrotizing Pancreatitis: An Evidence-Based Approach. Curr. Treat. Options Gastroenterol..

[38] Chantarojanasiri T., Ratanachu-Ek T., Isayama H. (2020). When Should We Perform Endoscopic Drainage and Necrosectomy for Walled-Off Necrosis?. J. Clin. Med..

[39] Chen Y.I., Yang J., Friedland S., Holmes I., Law R., Hosmer A., Stevens T., Franco M.C., Jang S., Pawa R. (2019). Lumen apposing metal stents are superior to plastic stents in pancreatic walled-off necrosis: A large international multicenter study. Endosc. Int. Open.

[40] Schmidt P.N., Roug S., Hansen E.F., Knudsen J.D., Novovic S. (2014). Spectrum of microorganisms in infected walled-off pancreatic necrosis—Impact on organ failure and mortality. Pancreatol. Off. J. Int. Assoc. Pancreatol. IAP.

[41] Kochhar R., Noor M.T., Wig J. (2011). Fungal infections in severe acute pancreatitis. J. Gastroenterol. Hepatol..

[42] Hoerauf A., Hammer S., Müller-Myhsok B., Rupprecht H. (1998). Intra-abdominal Candida infection during acute necrotizing pancreatitis has a high prevalence and is associated with increased mortality. Crit. Care Med..

[43] King N.K., Siriwardana H.P., Wood B., Siriwardena A.K. (2005). Trends in fungal colonization of pancreatic necrosis in patients undergoing necrosectomy for acute pancreatitis. HPB Off. J. Int. Hepato Pancreato Biliary Assoc..

[44] Werge M., Roug S., Novovic S., Schmidt P.N., Hansen E.F., Knudsen J.D. (2016). Fungal Infections in Patients With Walled-off Pancreatic Necrosis. Pancreas.

[45] De Waele J.J., Vogelaers D., Blot S., Colardyn F. (2003). Fungal infections in patients with severe acute pancreatitis and the use of prophylactic therapy. Clin. Infect. Dis. Off. Publ. Infect. Dis. Soc. Am..

[46] Kylänpää M.L., Repo H., Puolakkainen P.A. (2010). Inflammation and immunosuppression in severe acute pancreatitis. World J. Gastroenterol..

[47] Albers D., Meining A., Hann A., Ayoub Y.K., Schumacher B. (2021). Direct endoscopic necrosectomy in infected pancreatic necrosis using lumen-apposing metal stents: Early intervention does not compromise outcome. Endosc. Int. Open.

[48] Ryan B.M., Venkatachalapathy S.V., Huggett M.T. (2017). Safety of lumen-apposing metal stents (LAMS) for pancreatic fluid collection drainage. Gut.

